# Supplementary figures and images for: Delaying quantitative resistance to pesticides and antibiotics
Source: Evol Appl. 2022 Oct 25;15(12):2067–77. doi: 10.1111/eva.13497 (PMC9753825; doi:10.1111/eva.13497)

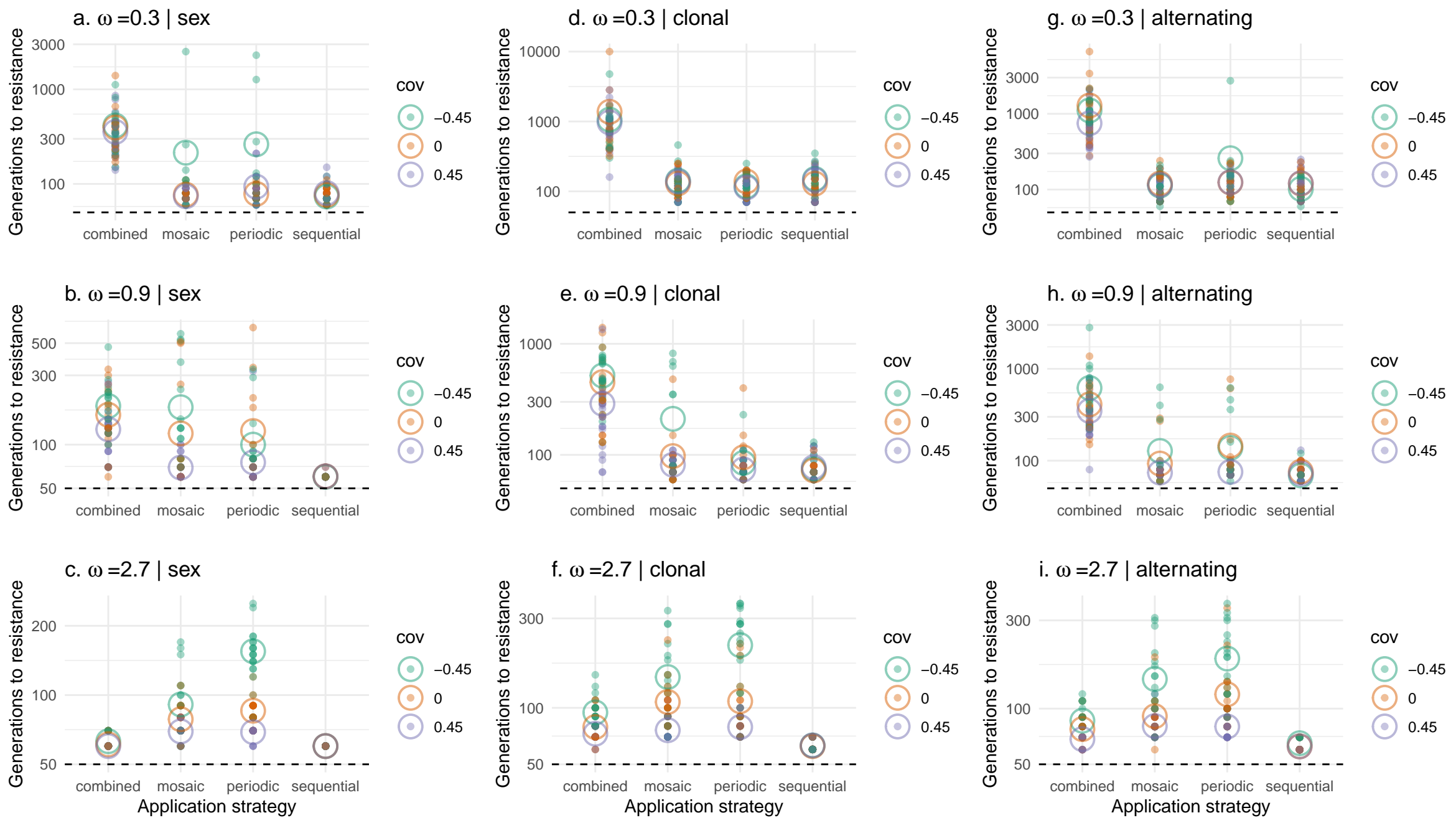

Supplement: Supplementary file 1 — Appendix S1 [file EVA-15-2067-s001.zip › eva13497-sup-0002-FigS2.pdf]

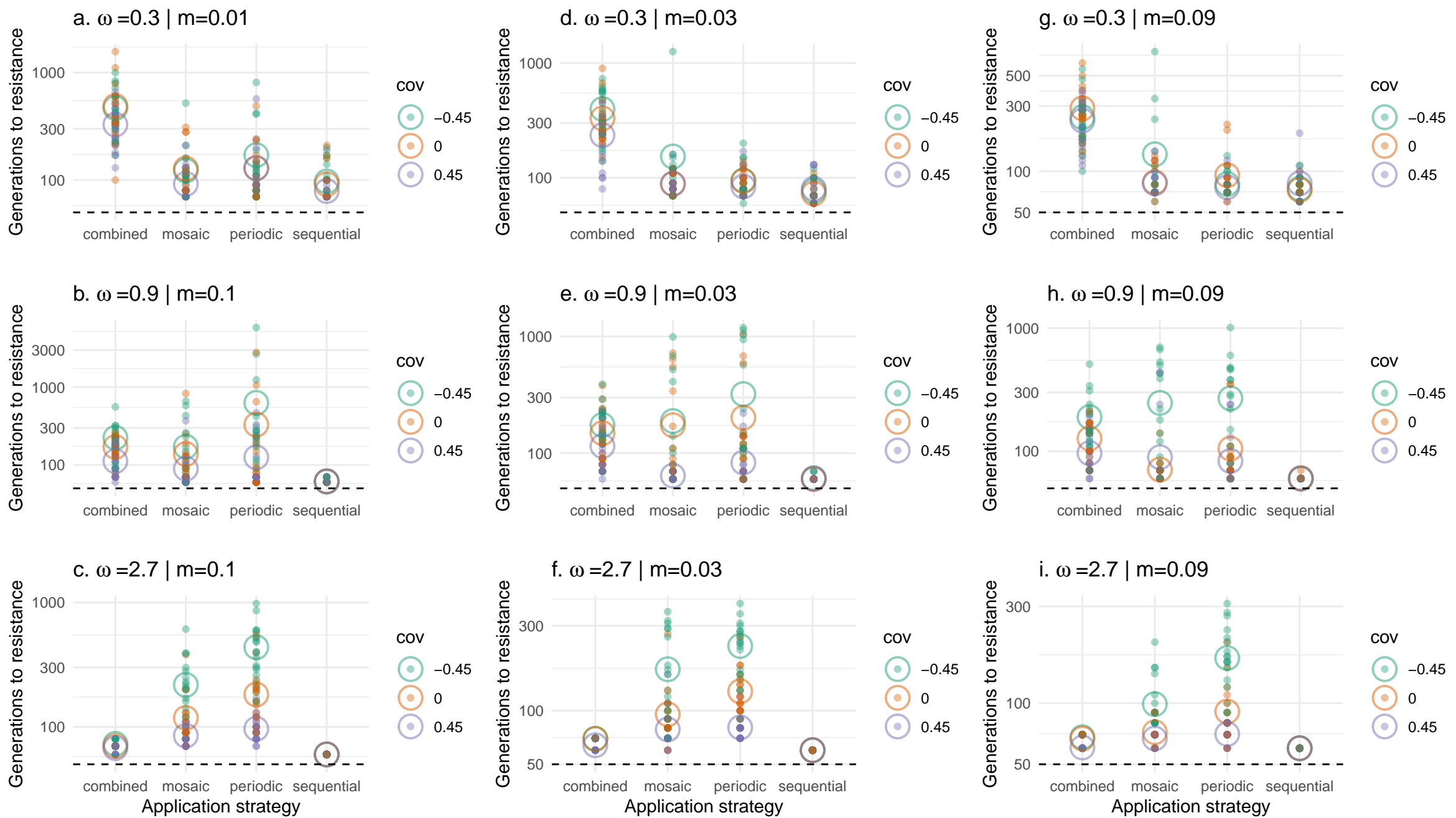

Supplement: Supplementary file 1 — Appendix S1 [file EVA-15-2067-s001.zip › eva13497-sup-0003-FigS3.pdf]

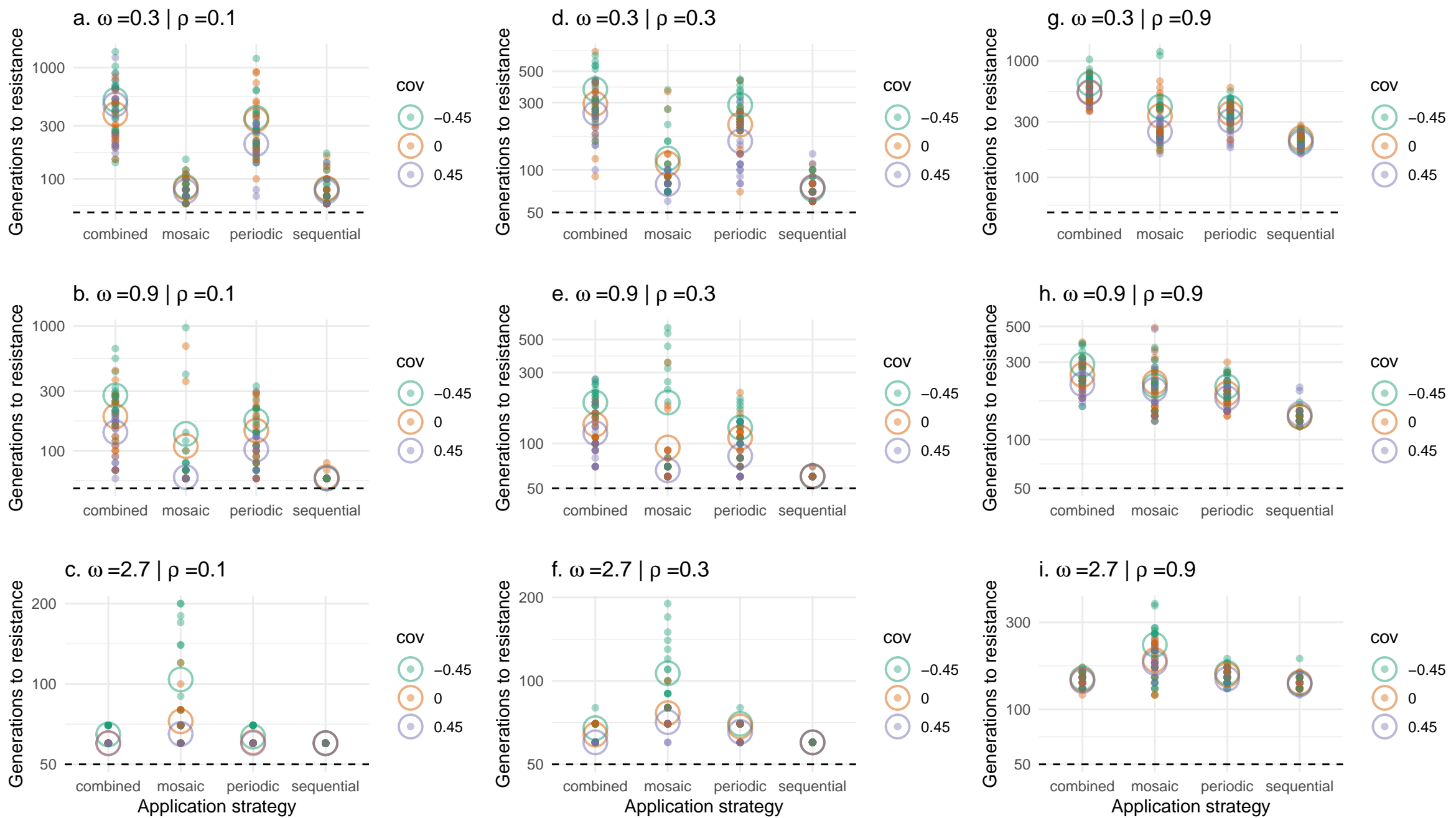

Supplement: Supplementary file 1 — Appendix S1 [file EVA-15-2067-s001.zip › eva13497-sup-0004-FigS4.pdf]

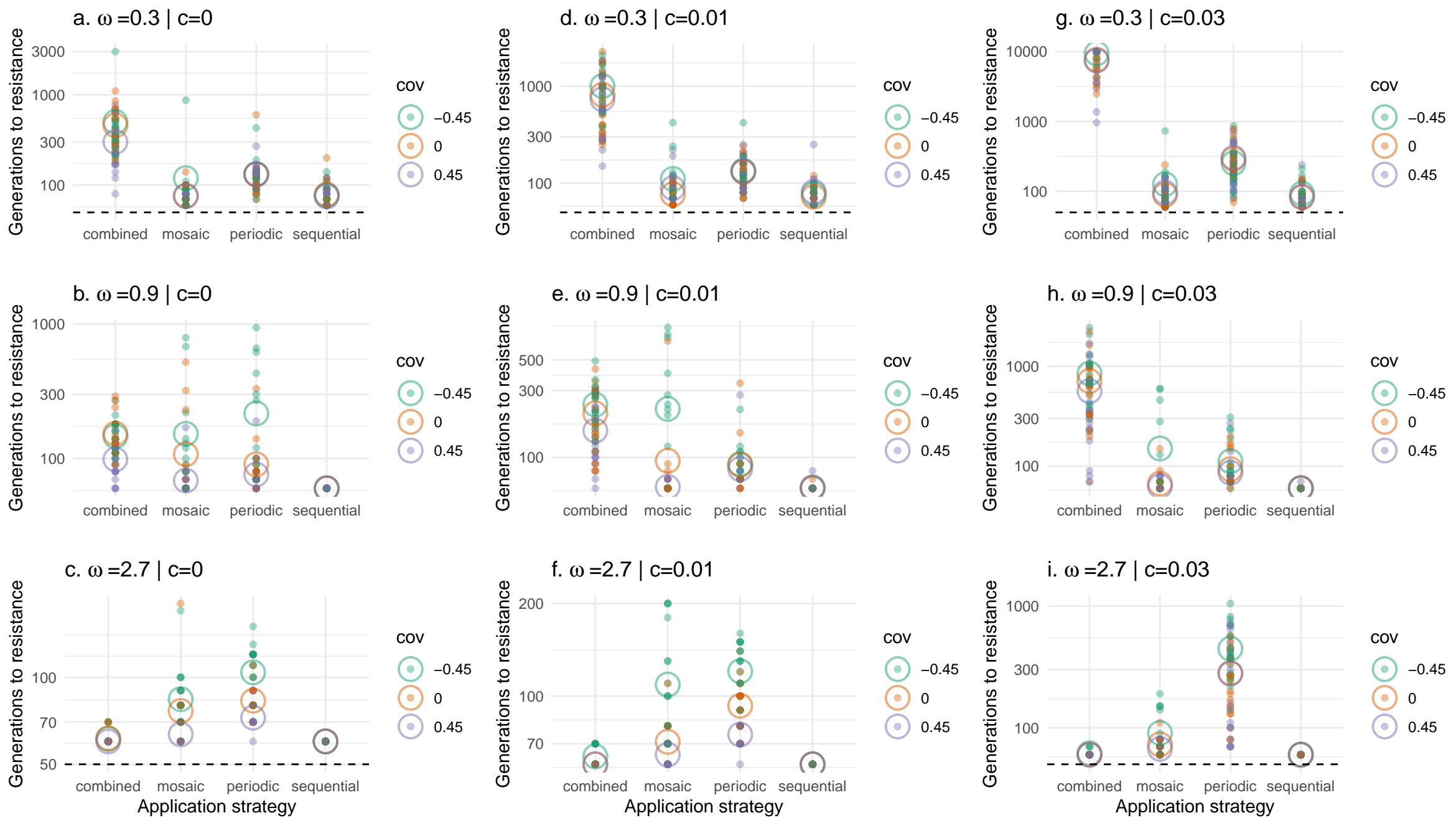

Supplement: Supplementary file 1 — Appendix S1 [file EVA-15-2067-s001.zip › eva13497-sup-0005-FigS5.pdf]
